# Laminoplasty with selective fusion at unstable segment versus laminectomy with fusion for multilevel cervical myelopathy: a case‐control study

**DOI:** 10.1186/s12891-021-04297-3

**Published:** 2021-05-07

**Authors:** Lin Du, Yanzheng Gao, Changqing Zhao, Tangjun Zhou, Haijun Tian, Kai Zhang, Jie Zhao

**Affiliations:** 1 Department of Spine Surgery, Henan Provincial People’s Hospital, People’s Hospital of Zhengzhou University, 7 Weiwu Road, 450000 Zhengzhou, Henan People’s Republic of China; 2 Shanghai Key Laboratory of Orthopedic Implants, Department of Orthopedics, Shanghai Ninth People’s Hospital, Shanghai Jiao Tong University School of Medicine, 639 Zhizaoju Road, 200011 Shanghai, People’s Republic of China

**Keywords:** Myelopathy, Segmental instability, Laminectomy and fusion, Laminoplasty, Selective fusion

## Abstract

**Background:**

Segmental cervical instability is a risk factor for the progression of osteophytic bone spurs and development of myelopathy, and is treated as a relative contraindication of cervical laminoplasty. The aim of this study was to compare laminoplasty with selective fixation (LPSF) versus laminectomy with fusion (LCF) in patients with multilevel cervical myelopathy accompanied by segmental instability.

**Methods:**

A case-control study was conducted by reviewing data from 63 patients who underwent LPSF (*n* = 30) or LCF (*n* = 33). Cervical alignment, range of motion (ROM), neurologic status and axial symptom severity pre-operation, 3-days after operation, and at the final follow-up (minimum 24 months) were measured and compared between groups.

**Results:**

Postoperation, patients in the LPSF group lost 31.1 ± 17.3 % of cervical lordosis and 43.2 ± 10.9 % cervical ROM while patients in the LCF group lost 5.7 ± 8.2 % and 67.9 ± 15.5 %, respectively. Both LPSF and LCF groups significantly improved neurologic status and axial symptom severity at the final follow-up with similar between-group results(*P* > 0.05). Blood loss, operation time, hospital stay, and medical cost in the LPSF group were significantly less than in the LCF group(*P* < 0.05).

**Conclusions:**

In 2 years of clinical observation, LPSF was effective in maintaining the stability of the cervical spine with less sacrifice of mobility and surgical trauma for multilevel myelopathy with segmental instability compared to LCF.

## Background

Multilevel degenerative changes and ossification of the posterior longitudinal ligament (OPLL) in the cervical spine leading to myelopathy has been studied extensively. Posterior decompression, including laminoplasty and laminectomy with fusion (LCF), are common procedures for multilevel spinal cord decompression.

Multilevel myelopathy often coexists with segmental dynamic instability, a degenerative change frequently seen in the elderly population. Segmental dynamic instability is a risk factor for the progression of osteophytic bone spurs and development of myelopathy [1, 2]. Laminoplasty is not an appropriate choice for this type of patient, because it can aggravate pre-existing segmental spinal instability which predisposes a patient to loss of cervical lordosis that may progress to kyphosis [3, 4]. Cervical kyphosis prevents indirect decompression and is associated with delayed neurologic deterioration and axial neck pain [5]. Therefore, laminectomy and fusion are commonly recommended for this group of patients. Despite posterior instrumentation to stabilize unstable segments and prevent post-laminectomy kyphosis, it has been recognized that without the protection of the lamina, adhesion formation may occur and lead to neurologic deterioration [6]. In addition, decrease in cervical range of motion (ROM) and higher incidence rate of C5 motor palsies [7] have been reported after LCF procedures.

Laminoplasty with fixation at the unstable segments was first reported by Yu et al. for the treatment of OPLL associated with segmental instability [8, 9]. Different from classic laminectomy with fusion and laminoplasty, this technique only fixes unstable segments after laminoplasty, while retaining the mobility of stable segments. Therefore, we call it laminoplasty with selective fixation at unstable segments (LPSF). This new technique has the potential to provide a stable environment for spinal cord recovery and prevents the progression of kyphosis and ossification, but also preserves the cervical ROM. However, there is a paucity of evidence-based comparisons between this promising new technology and standard LCF surgery. The current study addresses this gap, comparing outcomes between LPSF and LCF surgery for patients with identical operative indications in the hands of a single surgeon. The purpose of the present retrospective cohort study was to compare radiological and clinical outcomes of LPSF with LCF in the treatment of patients with multilevel cervical myelopathy accompanying segmental instability. We hypothesized that fixation at the unstable segments would not only maintain cervical sagittal alignment, but also preserve cervical motion, and then result in superior clinical outcomes than LCF surgery.

## Methods

### Patient selection

**Fig. 1:**
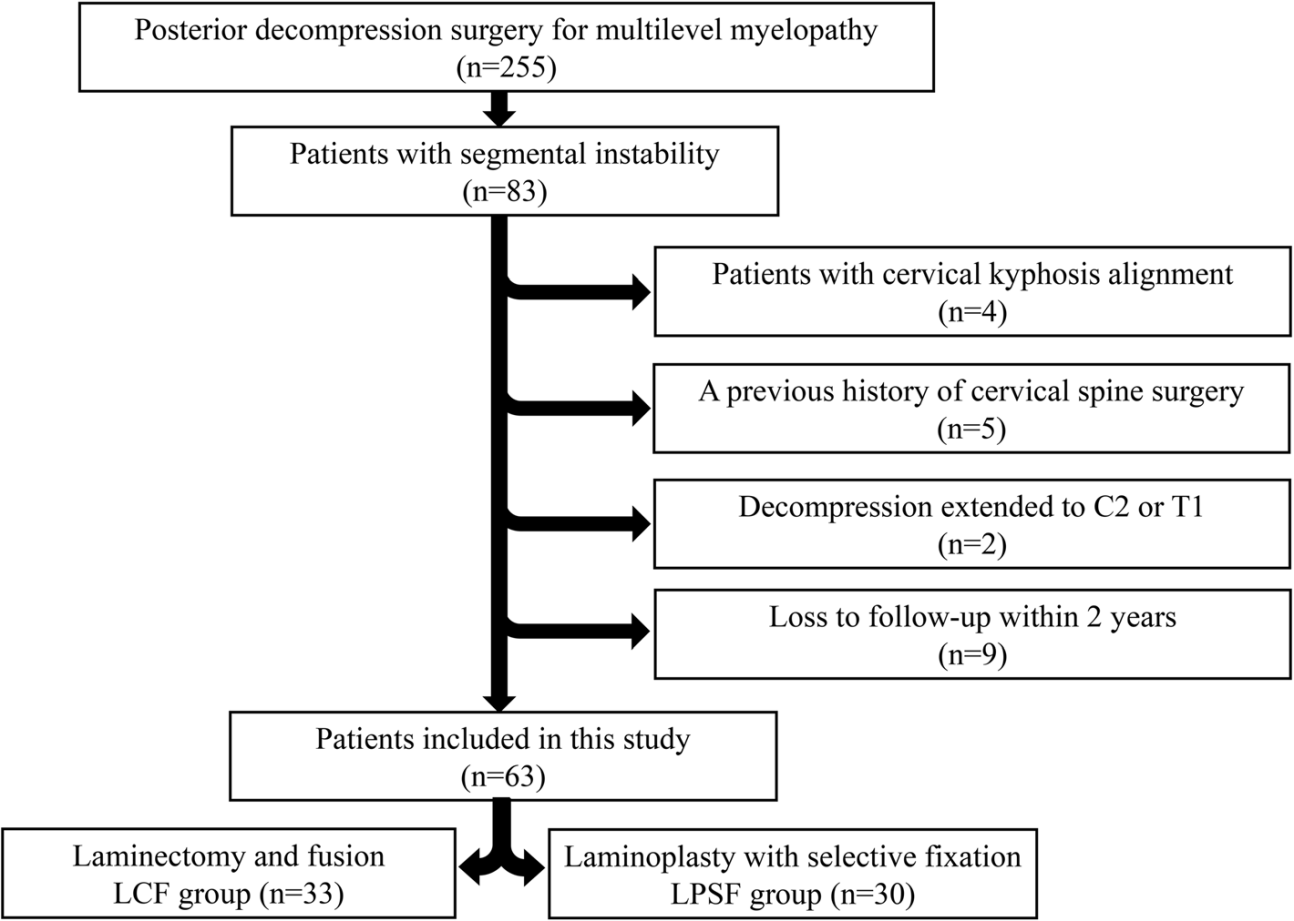
The flowchart of patient inclusion and exclusion

We reviewed medical records and radiological data of 255 patients who were diagnosed with cervical myelopathy and underwent posterior decompression surgery at Shanghai Ninth People’s Hospital between March 2010 and March 2015 (Fig. 1). We follow up patients in the outpatient clinic, and all patients take X-rays at each follow-up. The minimum follow-up time is 2 years.

Only patients with segmental dynamic instability were included in the study, which was confirmed by flexion-extension lateral radiography. The radiological criterion was defined as more than 3.5 mm horizontal displacement of one vertebra in relation to an adjacent vertebra or more than 11° change of segmental lordotic angle on pre-operation cervical dynamic lateral view radiography according to White and Panjabi standards [10].

**Fig. 2:**
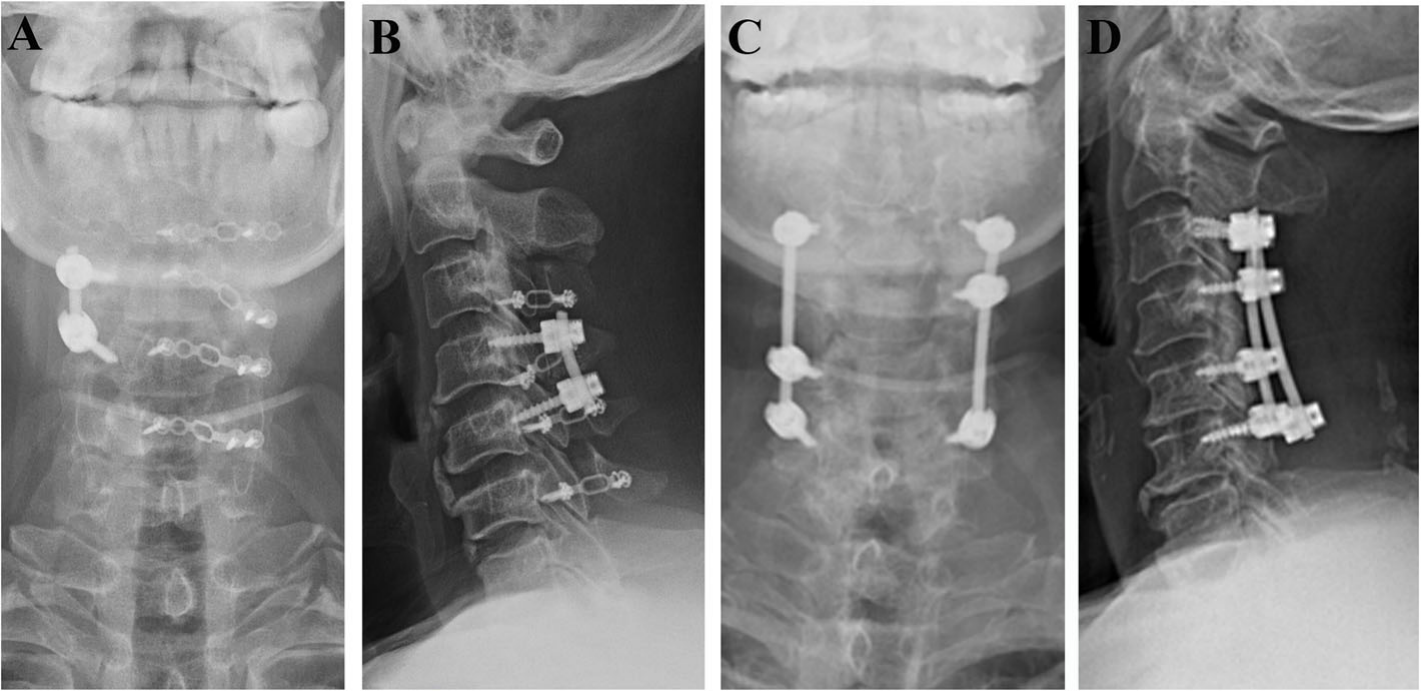
Postoperative cervical radiography of patients treated with laminoplasty and fixation at the unstable segment (**a** and **b**) and laminectomy and fusion (**c** and **d**)

Patients with cervical kyphosis alignment, a previous history of cervical spine surgery, decompression extended to C2 or T1, or loss to follow-up within 2 years after surgery were excluded from the study. The final study sample consisted of 63 patients with 30 patients in the LPSF group (Fig. 2a and b) and 33 patients in the LCF group (Fig. 2c and d). The LCF group was set as control group, because laminectomy and fusion are commonly recommended for multilevel myelopathy coexist with segmental instability. Laminoplasty was not set as control group, because Laminoplasty is not an appropriate choice for this type of patient. All patients included in the study showed symptoms such hand clumsiness, gait instability, loss of coordination, as well as spinal cord compression confirmed by magnetic resonance imaging (MRI) and computed tomography (CT) that could explain their symptoms.

### Surgical technique

For all patients, posterior decompression started at C3 and terminated at C6 or C7 and surgery was performed by the senior author (J.Z.). After induction of general anaesthesia, the patient was positioned prone in a prefabricated plaster cast. The unstable segment was confirmed by radiological findings. If LPSF was planned, the open side was placed on the side with the greater degree of symptoms. A laminoplasty plate was employed to support the lamina in a decompression position (Centerpiece, Medtronic Sofamor Danek, USA). Unilateral pedicle screw fixations were performed at the unstable segments. For these, the pedicle screws were inserted at the pedicle opposite to the side of dominance of the vertebral artery, regardless of whether the side was the open or hinge side. Autologous bone grafts were placed over the unstable segments on the hinge side. If LCF was scheduled, en bloc resection of the laminae and instrumentation with pedicle screw or lateral mass screw on the decompression segments were performed. For all patients, poly-axial screws combination with 3.5 mm titanium rods were used for posterior instrumentation (Vertex, Medtronic Sofamor Danek, USA). Postoperatively, all patients were taught to wear a Philadelphia collar for comfort and were encouraged to perform early neck muscle exercises.

### Radiographic evaluation

**Fig. 3:**
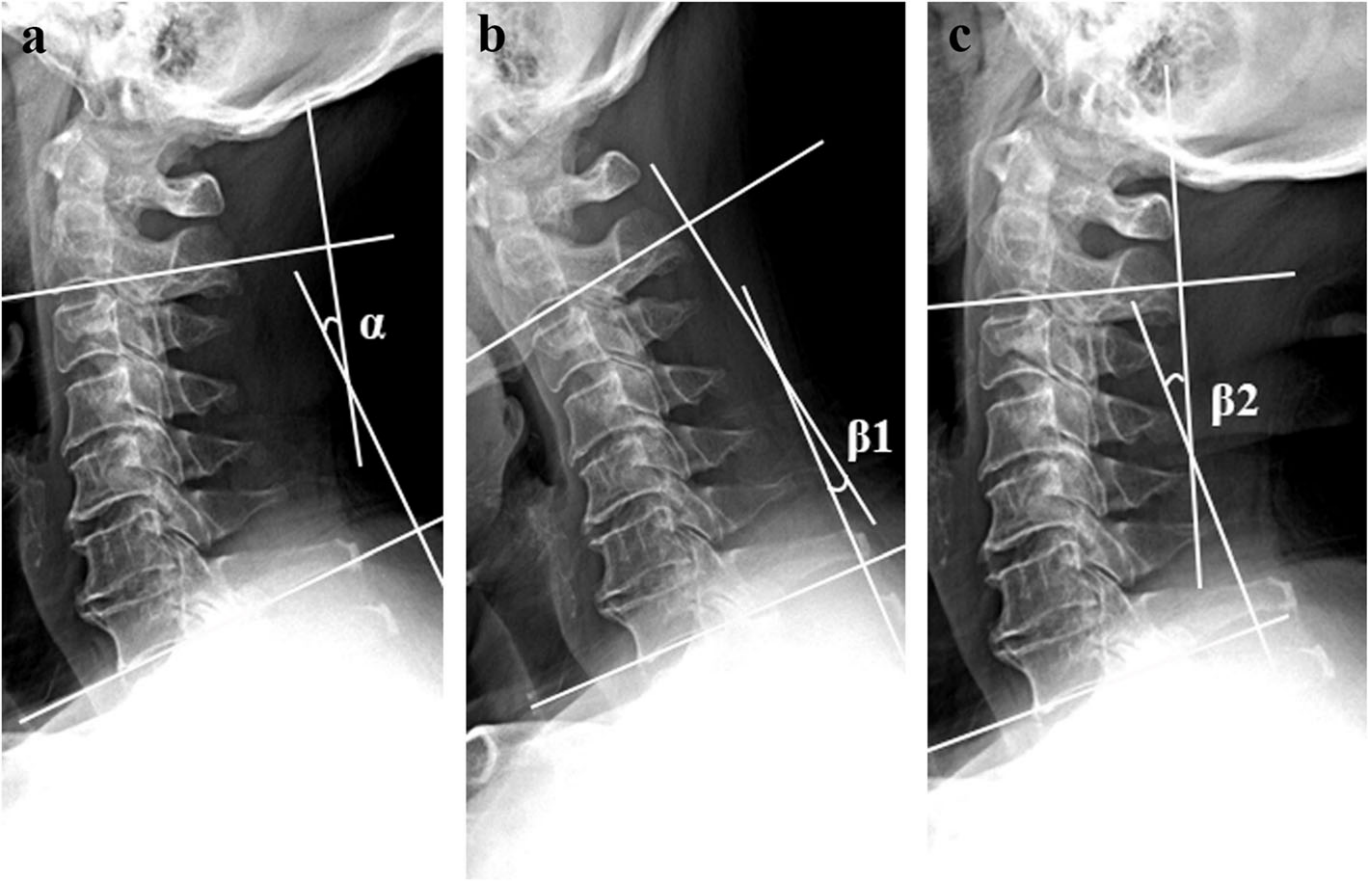
The C2-C7 Cobb (α) angle was defined as the angle between the line parallel to the inferior endplate of C2 and C7 (**a**). The cervical range of motion (ROM) was defined as the sum of C2-7 Cobb angles measured on flexion-extension lateral radiographs (**b** and **c**). ROM = β1 + β2

We evaluated the cervical alignment in upright standard lateral view radiographs obtained preoperatively, 3-days after operation, and at the final follow-up. Cervical sagittal alignment was assessed by the C2-7 Cobb angle. The C2-C7 Cobb angle was defined as the angle between the line parallel to the inferior endplate of C2 and C7 (Fig. 3a). Straight alignment was defined as fewer than 4˚ of either the kyphotic or lordotic angulations. Postoperative loss of cervical lordosis (LCL) was calculated with the following formula: LCL (%) = (3-day C2-7 Cobb angle - final C2-7 Cobb angle) /3-day C2-7 Cobb angle × 100 % [11].

Dynamic lateral views of the cervical spine taken before surgery and at the final follow-up were used to evaluate cervical ROM changes. We defined the cervical ROM as the sum of C2-7 Cobb angle measured on flexion-extension lateral radiographs (Fig. 3b and c) [12]. Loss of ROM (%) was calculated as (preoperative ROM - postoperative ROM) / preoperative ROM × 100 %. Measurements are performed using tools provided by the SYNAPSE Picture Archiving and Communication Systems. To evaluate and minimize interrater and intrarater difference, all radiologic images were evaluated 3 times by 2 of the authors (LD and CZ) independently and the mean value was used for analysis.

### Clinical evaluation

Clinical evaluation was made preoperatively and at the final follow-up. Neurological status was evaluated using the Japanese Orthopaedic Association (JOA) disability scale and axial symptom severity was quantified by the Visual Analogue Scale (VAS) and Neck Disability Index (NDI) [13]. The neurological recovery rate was calculated based on the following formula: recovery rate= (postoperative JOA score - preoperative JOA score) / (17 - preoperative JOA score) × 100 % [14]. Data on surgical parameters including duration, bleeding amount, length of hospital stay, and medical costs were also collected. Postoperative complications, including wound infection, instrumentation failure, cerebrospinal fluid (CSF) leak and nerve root palsy were recorded in detail.

### Statistical analysis

All continuous variables are reported as the mean ± standard deviation (SD) and categorical variables as frequencies and percentages. The Fisher’s exact test or chi-square test was used for comparison of categorical variables. For comparison of continuous variables between groups, we used independent sample t test if the assumptions of equal variance and the Gaussian distribution were met. Otherwise, a nonparametric Mann-Whitney test was used. To determine statistical significance of changes between preoperative and follow-up parameters in each group, we used paired t tests. Our missing data analysis procedures used missing at random (MAR) assumptions. We averaged estimates of the variables to give a single mean estimate and adjusted standard errors according to Rubin’s rules. Statistical analyses were performed using SPSS version 22.0 for Windows (SPSS, Inc., Chicago, IL, USA). p values < 0.05 based on a 2-sided hypothesis test were considered significant. The study power (1-β) for complications comparison was calculated with PASS 15 software (NCSS, LLC, Kaysville, Utah, USA).

## Results

### Baseline results

The general characteristics of patients are shown in Table 1. Of the 63 patients included in this study, the mean follow-up period was 29.0 ± 3.0 months in the LPSF group and 30.1 ± 3.7 in the LCF group. All of the 63 patients included in this study completed 2-year follow-up. No significant difference was detected among the groups in baseline demographic including sex (*p* = 0.597), age (*p* = 0.073), symptomatic duration (*p* = 0.085), follow-up period (*p* = 0.240), BMI (*p* = 0.189), Diabetes mellitus (*p* = 0.614), smoking (*p* = 0.182), and diagnosis (*p* = 0.556) (Table 1).

**Table 1:** Baseline patient demographics

Variables	Group LPSF (*n* = 30)	Group LCF (*n* = 33)	*p* value
Gender			0.597
Male (%)	21(70.0)	20(60.6)	
Female (%)	9(30.0)	13(39.4)	
Age (years)	61.1 ± 7.7	64.6 ± 7.4	0.073
BMI (kg/m^2^)	23.2 ± 4.2	24.5 ± 3.9	0.189
Smoker (*%)*)	7(23.3)	13(39.4)	0.182
Diabetes mellitus	7(23.3)	6(18.2)	0.614
Symptomatic duration (months)	39.1 ± 10.9	43.4 ± 8.9	0.085
Follow-up period (months)	29.0 ± 3.0	30.1 ± 3.7	0.240
Diagnosis			0.556
CSM	19	24	
OPLL	11	9	

*LPSF* laminoplasty and selective fixation at the unstable segment; *LCF  *laminectomy and fusion; *BMI* body mass index; *CSM* cervical spondylotic myelopathy; *OPLL* ossification of the posterior longitudinal ligament

### Radiological analysis

There were 25 one-segment and 5 two-segment fixation (1.2 segments fixed in average )in the LPSF group; and there were 10 three-segment and 23 four-segment fixation (3.7 segments fixed in average ) in the LCF group. The mean pre-operation C2-7 Cobb angle was 17.5 ± 7.7° in the LCF group and 17.4 ± 8.0° in the LPSF group. No patients with kyphosis cervical alignment were included in either group and the difference was not statically significant between the groups. After operation, both groups showed a slight increase in C2-7 Cobb angle. At the final follow-up the cervical alignment decreased by 5.7 % to 18.4 ± 6.9° in the LCF group and by 31.1 % to 13.4 ± 7.7° in the LPSF group. The change in sagittal alignment between the LCF and LPSF groups was statically different at the final follow-up(P < 0.001) (Table 2).

**Table 2:** Radiographic outcomes

Variables	Group LPSF (*n* = 30)	Group LCF (*n* = 33)	*p* value
C2-7 Cobb angle			
Preoperative (^o^)	17.4 ± 8.0	17.5 ± 7.7	0.941
3-day Postoperative (^o^)	18.1 ± 8.2	19.3 ± 6.0	0.528
Final (^o^)	13.4 ± 7.7	18.4 ± 6.9	0.013
loss of cervical lordosis (%)	31.1 ± 17.3	5.7 ± 8.2	< 0.001
Cervical ROM			
Preoperative (^o^)	32.7 ± 8.8	33.7 ± 8.4	0.647
Final (^o^)	19.2 ± 8.2	10.1 ± 3.7	< 0.001
loss of ROM (%)	43.2 ± 10.9	67.9 ± 15.5	< 0.001

*LPSF* laminoplasty and selective fixation at the unstable segment; *LCF* laminectomy and fusion; *ROM* range of motion

Preoperatively, the cervical ROM of the LPSF and LCF was 32.7 ± 8.8° and 33.7 ± 8.4°, respectively. At the final follow-up, both groups showed a significant reduction in ROM, however the magnitude of reduction was much larger in the LCF group 67.9 ± 15.5 % than in the LPSF group 43.2 ± 10.9 % (*p* < 0.001) (Table 2).

### Clinical analysis

There were no significant differences in preoperative self-reported clinical data between the 2 groups. Both groups (LPSF and LCF) showed significant improvement in their JOA, NDI and VAS scores postoperatively. The JOA score improved from 8.9 ± 2.3 (preoperative) to 14.1 ± 2.5 (postoperative) in the LPSF group and from 9.3 ± 2.5 (preoperative) to 14.5 ± 2.0 (postoperative) in the LCF group. The recovery rates of the JOA scores at the final follow-up was 66.8 ± 25.7 % in the LPSF group and 66.1 ± 26.9 % in the LCF group. No significant difference was detected in the final JOA score and JOA recovery rate between the 2 groups. Comparison of VAS and NDI score at the final follow-up showed that axial pain was not significantly different between the 2 groups (VAS: *p* = 0.722, NDI: *p* = 0.432) (Table 3).

**Table 3:** Clinical outcomes

Variables	Group LPSF (*n* = 30)	Group LCF (*n* = 33)	*P* value
JOA score			
Preoperative	8.9 ± 2.3	9.3 ± 2.5	0.481
Final	14.1 ± 2.5	14.5 ± 2.0	0.579
Recovery rate (%)	66.8 ± 25.7	66.1 ± 26.9	0.968
VAS score			
Preoperative	5.0 ± 2.2	4.2 ± 1.9	0.148
Final	1.6 ± 1.1	1.7 ± 1.4	0.722
NDI score			
Preoperative	17.3 ± 9.5	18.7 ± 6.9	0.495
Final	10.9 ± 5.6	11.9 ± 5.1	0.432
Blood loss (ml)	313.8 ± 88.4	387.5 ± 127.8	0.011
Operating time (min)	126.3 ± 27.1	145.4 ± 38.6	0.028
Length of stay (days)	5.8 ± 1.3	7.1 ± 1.5	< 0.001
Complications (n)			
Nerve root palsy	1	8	0.018
Superficial wound infection	2	3	1.000
CSF leakage	1	2	1.000
Instrumentation failure	0	0	NA
Reoperation	0	0	NA
Cost (RMB)	71.9 ± 11.2	78.6 ± 9.4	0.013

*JOA* Japanese Orthopaedic Association; *VAS* visual analogue scale; *NDI* neck disability index; *CSF* cerebrospinal fluid; *RMB* Chinese Yuan; *NA* Not Applicable

In the LCF compared with LPSF group, there were longer operating times, greater amount of blood loss, longer hospital stays, and higher medical costs, and the differences were statistically significant. There were 1 nerve root palsy in the LCF group and 8 in the LPSF group. Based on previous reports, nerve root palsy occurs in 5–16 % of patients undergoing posterior decompression[15–17], the power with regard to nerve root palsy was calculated. However, with a sample size of 30 subjects in the LPSF group and 33 subjects in the LCF group, the current study has only 12.9 % power to detect the difference of nerve root palsy occurrence between two groups. The incidence of superficial wound infection and CSF leakage were low in both groups. Spinal fusion was noted in 100 % of patients at the last follow-up. No instrumentation failure or reoperation was reported in this group of patients (Table 3).

## Discussion

The purpose of the present study was to compare radiological and clinical outcomes of LPSF with LCF in the treatment of patients with multilevel cervical myelopathy accompanying segmental instability. Our results suggest that LPSF was effective in maintaining the stability of the cervical spine with less sacrifice of mobility and surgical trauma for multilevel myelopathy with segmental instability compared to LCF.

Well maintained cervical alignment is needed for indirect spinal cord decompression. It is intuitive that loss of cervical lordosis is especially relevant to the LPSF group but not to the LCF group. This is manifested within the present study where the cervical lordosis was well maintained in the LCF group. Meanwhile, in the LPSF group there was a 31.1 % decreased of cervical lordosis and 5 patients developed straight alignment at the final follow-up. This result is similar to pervious reports of cervical lordosis loss after laminoplasty surgery alone [4]. Because all patients in this study have segmental instability, which predisposes a patient to loss of cervical lordosis that may progress to kyphosis, this result indicates that LPSF contribute to segments stabilization, and decreases cervical lordosis loss. Considering it is possible that LPSF may result in loss of lordosis and even progression to kyphosis, same as laminoplasty alone technique, LPSF should only be recommended for patients with a well-aligned cervical spine.

Restricted cervical motion after operation is one of the patient concerns before cervical spinal operations. Preserving cervical movement is an advantage of laminoplasty over laminectomy and fusion. With strong metal instrumentation, LCF is a powerful tool to correct mal-aligned cervical spines and maintain cervical lordosis, but much of the cervical spine mobility is scarified. There is also a reduction of cervical ROM after laminoplasty alone, assumed to be caused by spontaneous laminar fusion [18]. Therefore, it is meaningful to investigate the effect of additional selective fusion after laminoplasty and its effect on cervical ROM. In this study, we found both groups experienced a significant decrease in ROM, but the decrease was considerably less in the LPSF than in the LCF group. This result indicates that LPSF provides the benefit of preserving physiological cervical motion.

Preoperative cervical kyphosis is not suitable for cervical laminoplasty and loss of cervical lordosis is correlated with delayed neurological function deterioration [19]. In this study, although LPSF was less powerful than LCF in preventing loss of cervical lordosis, this effect did not result in inferior neurological recovery. We found that both LPSF and LCF significantly improved the JOA score and no significant difference was detected in the recovery rates between the 2 groups. These results were similar to previous reports on the comparison between lanimoplasty and LCF [20].

Axial symptoms, including neck and shoulder pain and shoulder muscle spasm, occur frequently in patients with cervical spondylosis and have a significant negative impact on quality of life [21]. The axial symptoms are generally considered to originate from degenerated intervertebral discs or facet joints and are reported to worsen or newly emerge after laminoplasty [22]. Segmental instability is a significant risk factor for neck pain after laminoplasty [23, 24] and postoperative cervical ROM restriction could alleviate segmental instability and concomitant axial symptoms [24, 25]. In this study, both LPSF and LCF patients showed significant improvement in VAS and NDI scores at the final follow-up and the improvement between the 2 groups was similar. These findings indicate that selective fusion is effective for relieving axial symptoms after laminoplasty due to stabilization at the unstable segments and/or the restriction of cervical ROM after LPSF.

Extensive posterior structure resection and instrumentation will increase surgical time, blood loss and medical expenditure. As shown in this study, LPSF resulted in less surgical time and blood loss, indicating a relatively less invasive technique. Because implant costs are a big part of medical expenditure for patients in China, LPSF was more cost effective than LCF. We noticed shorten hospital stays may have also decreased the expenditure of patients who underwent LPSF surgery.

Due to the low incidence of complications of both technologies, a large sample size is needed to detect the difference of complications. Therefore, a limitation of this study is the relatively small simple size. LPSF preserved the lamina, which may restrict excessive spinal cord posterior drift at the level of C5 and decreased mechanical tethering of C5 nerve root [26]. However, due to the limited sample size of the current study, it was not enough to support a lower incidence of C5 nerve root palsies in the LPSF group. The incidence of surgical complications were low in both groups, including wound infection, instrumentation failure, and CSF leakage. This study did not provided enough power to support significant difference of incidence of these complications either. Larger sample size research is needed in the further study.

There are other limitations to consider in the present study. We acknowledge the grouping was not prospectively randomized and controlled. Nearly all the LCFs were performed in the first two years, and LPSFs in the last four years of the study period. This variation was based on new techniques for less invasive and effective treatment of patients, but could have introduced bias. However, the demographic and baseline outcomes between the 2 groups were similar. We believe that the well-matched grouping was the major strength of this study. Furthermore, the follow-up time for both groups are limited. A follow-up of a few years (5 years or more) would be more adequate to see the longer term complications of LPSF and LCF. Considering the limitation of the current study, the efficiency of LPSF needs to be determined by further long-term clinical observation.

## Conclusions

In conclusion, this 2-year clinical study suggest that LPSF produces comparable clinical outcomes with LCF surgery in the treatment of patients with OPLL accompanied by segmental instability. LPSF was found to be superior to LCF in preserving cervical ROM and decreasing surgical trauma and medical cost.

## Data Availability

The datasets used and/or analysed during the current study are available from the corresponding author on reasonable request.

## Abbreviations

CSF: Cerebrospinal fluid
CT: Computer tomography
JOA: Japanese orthopaedic association
LCF: Laminectomy with fusion
LCL: Loss of cervical lordosis
LPSF: Laminoplasty with selective fixation
MRI: Magnetic resonance imaging
NDI: Neck disability index
OPLL: Ossification of the posterior longitudinal ligament
ROM: Range of motion
VAS: Visual analogure scale
